# Motor, Somatosensory, Viscerosensory and Metabolic Impairments in a Heterozygous Female Rat Model of Rett Syndrome

**DOI:** 10.3390/ijms19010097

**Published:** 2017-12-29

**Authors:** Aritra Bhattacherjee, Michelle K. Winter, Linda S. Eggimann, Ying Mu, Sumedha Gunewardena, Zhaohui Liao, Julie A. Christianson, Peter G. Smith

**Affiliations:** 1 Department of Molecular and Integrative Physiology, University of Kansas Medical Center, Kansas City, KS 66160, USA; Aritra.Bhattacherjee@childrens.harvard.edu (A.B.); mu2@uthsc.edu (Y.M.); sgunewardena@kumc.edu (S.G.); zliao@kumc.edu (Z.L.); 2 Kansas Intellectual and Developmental Disabilities Research Center, University of Kansas Medical Center, Kansas City, KS 66160, USA; mwinter2@kumc.edu (M.K.W.); leggimann@kumc.edu (L.S.E.); jchristianson@kumc.edu (J.A.C.); 3 Department of Anatomy and Cell Biology, University of Kansas Medical Center, Kansas City, KS 66160, USA

**Keywords:** autism spectrum disorder, behavior, feeding

## Abstract

Rett Syndrome (RTT), an autism-related disorder caused by mutation of the X-linked Methyl CpG-binding Protein 2 (*MECP2*) gene, is characterized by severe cognitive and intellectual deficits. While cognitive deficits are well-documented in humans and rodent models, impairments of sensory, motor and metabolic functions also occur but remain poorly understood. To better understand non-cognitive deficits in RTT, we studied female rats heterozygous for *Mecp2* mutation (*Mecp2^−/x^*); unlike commonly used male *Mecp2^−/y^* rodent models, this more closely approximates human RTT where males rarely survive. *Mecp2^−/x^* rats showed rapid, progressive decline of motor coordination through six months of age as assessed by rotarod performance, accompanied by deficits in gait and posture. *Mecp2^−/x^* rats were hyper-responsive to noxious pressure and cold, but showed visceral hyposensitivity when tested by colorectal distension. *Mecp2^−/x^* rats ate less, drank more, and had more body fat resulting in increased weight gain. Our findings reveal an array of progressive non-cognitive deficits in this rat model that are likely to contribute to the compromised quality of life that characterizes RTT.

## 1. Introduction

Autism spectrum disorders (ASDs) and related ASD-like disorders are recognized primarily by cognitive and intellectual deficits. Rett syndrome (RTT) is one of the most severe autism-like disorders affecting, one in 10,000 female births. It is caused by mutation of the X-linked gene *MECP2*, which is highly expressed in neurons and regulates chromatin organization and global gene transcription [1,2,3]. RTT is associated with severe cognitive, psychomotor and intellectual disabilities [4,5]. Intellectual and cognitive defects in RTT have been extensively studied in humans as well as animal models [6]. Cognitive deficits such as impaired sociability, anxiety, nest building, contextual fear conditioning, memory function and behavioral regression are prominent in rodent models [7,8,9,10,11,12,13,14], and studies using these *MECP2* knockout animals have contributed much to our current mechanistic understanding of RTT [15].

RTT subjects are also reported to exhibit profound deficits in a wide range of basic physiological parameters including sensory perception, motor coordination, respiration and GI function, as well as metabolic processes [4,5,16,17,18,19,20]. These dysfunctions contribute to reduced quality of life and health, and frequently aggravate behavioral symptoms. The nature of these dysfunctions and the time course over which they appear, however, remain poorly understood. It has been particularly difficult to document these features in human subjects, given that communication skills are severely compromised by this disability. Unlike other ASDs whose origins involve complex gene–environment interactions or are unidentified, RTT is monogenic and mutation of the *MECP2* gene alone in animal models recapitulates many features of the human disease. This provides an opportunity to systematically study onset, nature and progression of non-cognitive dysfunctions [4,6] in RTT.

RTT in humans is essentially a female disorder. While both males and females may harbor *Mecp2* mutations of the X chromosome, males born with the mutation typically die within a few months due to severe encephalopathy [4]. Hence, more than 90% of surviving human RTT patients are heterozygous females with *Mecp2* mutation (*Mecp2^−/x^*) [4]. Females with RTT display mosaicism in the expression of the *Mecp2* mutation; X chromosome inactivation leads to normal expression of *Mecp2* in some cells while others lack expression. However, animal studies to date have relied predominantly on male hemizygous rodents (*Mecp2^−/y^*). Male rodent knockouts (KO) survive after birth but display a more severe phenotype that fails to capture the regressive stages of the disease that occur in humans and show a reduced lifespan of up to 2 months [4,6,15], whereas human females can typically live to middle age. Accordingly, limitations of the standard male rodent model of RTT make it desirable to explore models that may more faithfully capture features of the disorder.

In the current study, we used a heterozygous female rat (*Mecp2^−/x^*) that more closely approximates human RTT, and studied longitudinally a wide range of non-cognitive functions.

## 2. Results

### 2.1. Motor Function

**Figure 1:**
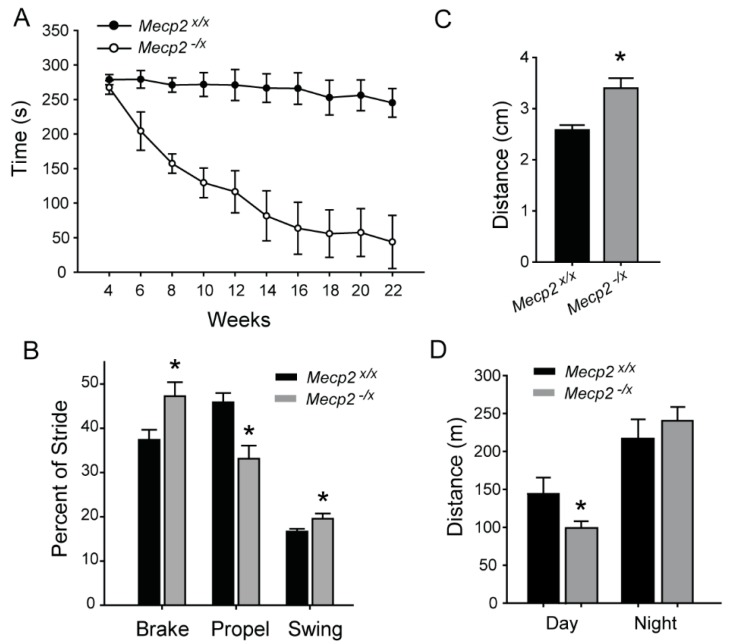
Motor impairments in RTT. (**A**) Biweekly measurements of Rota-rod performance in WT *Mecp2^x/x^* and *Mecp2^−/x^* rats. Two-way repeated measure Analysis of Variance (ANOVA) reveals significant difference at all ages starting from six weeks (*p* < 0.001); Holm-Sidak post hoc comparisons (*n* = 10 per group). See Table 1 for statistical details; (**B**) Forepaw gait analysis using the DigiGait system in WT and *Mecp2^−/x^*. Comparison by *t*-test reveals significant difference between the two groups in Brake (* *p* = 0.021), Propel (* *p* = 0.0027) and Swing (* *p* = 0.024), expressed as percentage of Stride, as measured from the forepaw; (**C**) Hind paw stance width of the mutant animals was greater (* *p* = 0.0009, *t*-test). *n*: *Mecp2^x/x^* =10 and *Mecp2^−/x^* = 6 for (**B**) and (**C**); 4 *Mecp2^−/x^* animals were excluded because they could not stay on the DigiGait treadmill; (**D**) Difference in distance traveled in a 24-h period by the rats, split between the day and night cycle. Mutant rats are hypoactive during the day (* *p* = 0.037), but show no difference relative to wildtype at night (*p* = 0.434) (*n* = WT = 5 and *Mecp2^−/x^* = 10). All graphs represent Mean ± SEM.

Motor deficits are one of the most prominent non-cognitive impairments in girls with RTT [4]. We assessed motor coordination by rotarod performance test in *Mecp2^−/x^* rats longitudinally over time. Starting at four weeks of age, we measured biweekly the ability of *Mecp2^−/x^* and *Mecp2^x/x^* rats to remain on the rotarod during a progressively increasing rate of rotation. There was no difference between groups at four weeks, and *Mecp2^x/x^* showed consistent performance through 22 weeks (Figure 1A). However, biweekly measurements of *Mecp2^−/x^* showed significant declines at 6–10 weeks, plateauing with severely impaired performance at around 16 weeks (Figure 1A and Table 1).

To gain further insight into the mechanics of locomotor dysfunction, we analyzed gait at 22 weeks, after the decline in rotarod performance had reached a stable plateau. DigiGait parameters for gait analysis can be broken down into the major phases of the step cycle, “brake”, “swing” and “propel” [21]. “Brake” is the interval from the first contact with the surface to peak stance, “Propel” is the phase from peak until end of contact, and the transitional phase during which a paw is not in contact and extends forward is referred to as “swing”. In the forepaw step cycle, brake and swing times were increased, whereas time spent in the propel phase was decreased relative to wild type (WT) rats (Figure 1B). In contrast, the hind paw step cycle did not show statistically significant differences (not shown); however, hind limb stance width, which is the distance between the centers of the hind paws at peak stance, was increased, reflecting postural deficits (Figure 1C). *Mecp2^−/x^* also showed forepaw ataxia; the ataxia coefficient (maximum stride length minus minimum stride length) in *Mecp2^x/x^* = 1.6 ± 0.093, and *Mecp2^−/x^* = 1.24 ± 0.129 (*p* = 0.038).

Because alterations in motor control of stepping could impact ambulation, we assessed free ambulatory behavior of the rats. Measurements over a 24 h period at 22 weeks revealed a moderate decline of diurnal activity. However, there was no difference in nocturnal activity in WT and mutant rats (Figure 1D).

### 2.2. Sensory Function

Because studies in both humans and male rodent models of RTT suggest that sensory function is altered [16,17,22,23], we studied responses to somatosensory stimuli including mechanical pressure, noxious heat and noxious cold. Behavioral responses to these stimuli were measured longitudinally from four through 22 weeks of age.

**Figure 2:**
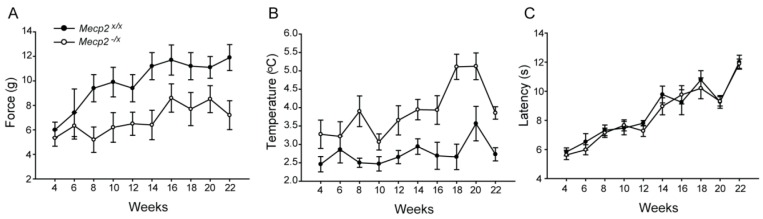
Somatic sensitivity in the RTT rat model. (**A**) Biweekly measurements using von Frey monofilaments reveals mechanical hypersensitivity in *Mecp2^−/x^* rats at all ages starting from eight weeks (Holm-Sidak post hoc comparisons, *p <* 0.001 at 8–22 weeks); (**B**). Biweekly measurements of noxious cold sensitivity using the cold plate reveals hypersensitivity in *Mecp2^−/x^* rats at all ages starting from 8 weeks (two-way repeated measure ANOVA, Holm-Sidak post hoc comparisons, *p <* 0.001); (**C**) Noxious heat sensitivity tested using the Hargreaves Paw Thermal Analgesiometer. No difference between the two groups registered during the entire testing period. (*n*: WT = 10, *Mecp2^−/x^* = 14). Further details of statistical parameters for (**A**,**B**) in Table 1. All graphs represent Mean ± SEM.

Mechanical sensitivity was tested using graded von Frey monofilaments applied to the hind foot paw to determine the threshold for withdrawal response. Thresholds were generally lower in younger rats and no significant difference could be resolved between wild type and heterozygotes at four and six weeks of age. However, while thresholds rose beginning at eight weeks in *Mecp2^x/x^* as part of normal developmental course, *Mecp2^−/x^* rats remained hypersensitive throughout the adult life with little change of threshold through 22 weeks (Figure 2A and Table 1).

Response to noxious cold was tested by recording the temperature for paw withdrawal on a cold plate. A general tendency toward higher sensitivity to noxious cold existed in *Mecp2^−/x^* rats from the beginning; however, significant differences became prominent from eight weeks of age that grew more severe over the subsequent weeks of testing (Figure 2B and Table 1).

Response to noxious heat was tested by using the paw thermal analgesiometer, where a light beam is directed to the Plexiglas surface immediately beneath a hind paw and the latency for foot withdrawal is measured. Both WT and *Mecp2^-/x^* rats showed increased response latencies with age, consistent with a developmental diminution in thermal sensitivity. However, unlike mechanical or cold sensitivity, there were no differences in response latencies to noxious heat between the two groups at any age point (Figure 2C).

**Figure 3:**
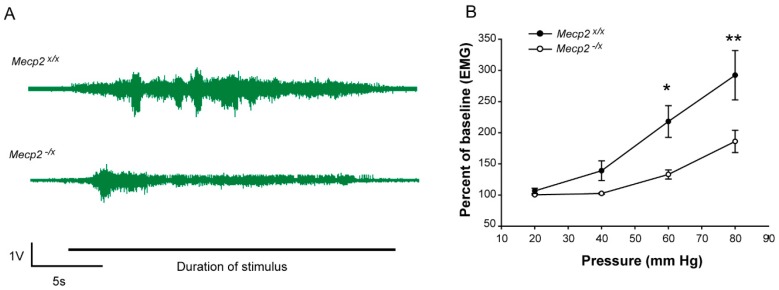
Visceral sensitivity in the RTT rat model. (**A**) Representative electromyography (EMG) recordings of visceromotor response in abdominal muscles of *Mecp2^x/x^* and *Mecp2^−/x^* rats for colorectal distension at 80 mm Hg; (**B**) Comparison of visceromotor responses expressed as activity per unit time, in 22-week-old rats reveal hyposensitivity to colorectal distension at 40, 60 and 80 mm Hg pressures. EMG/s in *Mecp2^−/x^* rats is significantly lower at 60 mm Hg (* *p* = 0.006) and 80 mm Hg (** *p <* 0.001) pressures (two-way repeated measure ANOVA, Holm–Sidak Posthoc Analysis; *n*: WT = 10, *Mecp2^−/x^* = 8. See Table 1 for details. Graph represents Mean ± SEM.

The gastrointestinal system is imbued with stretch receptors that monitor pressure and elicit reflex contractile responses in the surrounding musculature [24]. Because there are reports that RTT patients show visceral discomfort under certain conditions, we assessed whether responses to colorectal distension [24] are affected in the *Mecp2^−/x^* rat. (Figure 3A). In response to applied colorectal pressures from 20 to 80 mm Hg, the visceromotor response (VMR) increased in both WT and *Mecp2^−/x^* animals. However, the heterozygous animals showed reduced VMR relative to WT rats at pressures from 40 to 80 mm Hg (Figure 3B and Table 1), consistent with visceral hyposensitivity.

### 2.3. Metabolic Indicators

**Figure 4:**
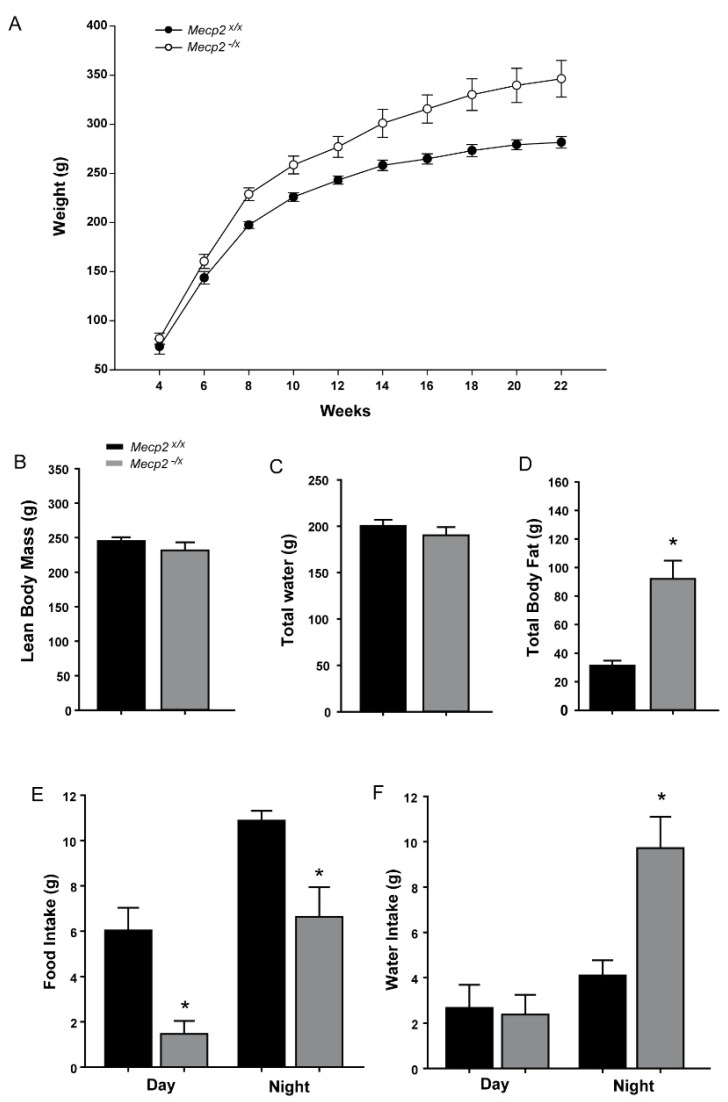
Abnormal weight gain and calorie intake in the RTT rat model. (**A**) Biweekly weight measurements from *Mecp2^x/x^* and *Mecp2^−/x^* rats reveal a significantly increased body weight starting from eight weeks of age (*p <* 0.001, two-way repeated measure ANOVA with Holmes-Sidak posthoc analysis; see Table 1 for statistical details); (**B**–**D**) Comparison of body mass composition between *Mecp2^x/x^* and *Mecp2^−/x^* rats by Echo-MRI analysis. No difference in lean body mass (**B**) or total water content (**C**) detected between the two groups. Body fat levels were highly elevated in the *Mecp2^−/x^*; (**D**) *p* = 0.0001; (**E**) Food intake comparison between the two groups revealed lower consumption by the *Mecp2^−/x^* rat both during day (*p* = 0.001) and night (*p* = 0.044) (*t*-tests); (**F**) Water intake compared between the two groups of rats. No diurnal difference, but nocturnal consumption is highly increased in *Mecp2^−/x^* rats (*p* = 0.016, *t*-test. *n*: WT = 5, *Mecp2^−/x^* = 10). All graphs represent Mean ± SEM.

Metabolic dysfunction is increasingly associated with autism spectrum disorders [25,26]. We therefore assessed indices of metabolic activity to determine if female rats heterozygous for the *Mecp2* mutation showed phenotypes consistent with this type of abnormality. At postnatal weeks 4–6, body weights of *Mecp2^−/x^* and *Mecp2^x/x^* rats were comparable. Beginning at eight weeks of age, however, *Mecp2^−/x^* rats were heavier and the difference increased with time throughout the 22 weeks of the study (Figure 4A).

We assessed the proximate cause of the increased body weight by analyzing body mass composition at 22 weeks using an Echo-MRI. We found no difference in the lean body mass or total body water content between the two groups (Figure 4B,C). However, total body fat was elevated approximately three-fold in the *Mecp2^−/x^* relative to the *Mecp2^x/x^* rats (Figure 4D).

To assess whether there may be strain-dependent differences in caloric intake, we measured food consumption over a 48-h period in a metabolic chamber. In WT rats, food was consumed in the diurnal phase although nocturnal consumption was significantly greater (Figure 4E). Surprisingly, *Mecp2^−/x^* rats ate substantially less food (Figure 4E). Night time consumption was reduced by approximately 1/3, whereas consumption during the daytime was only about 30% of WT controls.

Water intake was also affected by the *Mecp2* mutation. WT rats showed similar water consumption in both the diurnal and nocturnal phases (Figure 4F). Daytime drinking in *Mecp2^−/x^* rats was similar to WT; however, they drank approximately twice as much water at night relative to WT rats (Figure 4F).

To assess whether changes in metabolic rate may help to explain why *Mecp2^−^^/x^* rats gain weight and fat mass despite lower food consumption, we assessed a range of metabolism-associated parameters (Table 2). *Mecp2^−^^/x^* rats showed consistently lower metabolic values, with a significantly lower respiratory quotient detected during the light phase of the diurnal cycle.

### 2.4. Inter-Relationships among MECP2 Mosaicism and Behavioral and Metabolic Dysfunctions

Because *MECP2* is an X-linked gene, random X-chromosome inactivation means that each RTT female patient (or rat) is a mosaic of *MECP2* expressing and null cells, and the degree of mosaicism among different individuals can vary. We reasoned that the degree of mosaicism, or heterozygosity, may correlate with disease severity. We analyzed *Mecp2^−/x^* rats for their degree of mosaicism in peripheral blood T-cells as a representative cell population that is easily isolated. Blood samples were drawn from tail veins of the rats and peripheral blood mononuclear cells (PBMNCs) isolated. PBMNCs were fixed and stained for *Mecp2* and the T-cell marker CD3. Fluorescence assisted cell sorting (FACS) analysis of these samples revealed the percentage of T-cells expressing *Mecp2*. We were unable to detect any significant correlation between degree of T-cell mosaicism and behavioral or metabolic dysfunction in any of the measured parameters. Similarly, when we assessed the relationships among different behavioral and metabolic parameters, strong correlations were not evident with the exception of deficits in rotarod performance correlating with a decrease in the swing phase of the step cycle (Table 3).

## 3. Discussion

This work demonstrates the feasibility of using heterozygous female rats to study progressive non-cognitive pathologies associated with RTT. While homozygous male *MECP2* mutant rodents have been used for most RTT animal model studies to date, almost all human patients are heterozygous females. Our observations show that heterozygous female animals develop impairments similar to those seen with homozygous males [22], albeit with later onset, and potentially greater variability that is consistent with human observations. Moreover, the rat has proven advantageous as a model for resolving the longitudinal progression of disease symptoms. Behavioral responses in rat are robust and provide a broad dynamic range to detect regressive changes over time. In fact, before the advent of mouse genetics, the rat was a highly preferred model for study of sensory, motor and cognitive behaviors [27].

Consistent with the human disorder [28], motor impairments emerged as one of the most significant deficits in the *Mecp2^−/x^* rats. Motor behavioral deficits have been reported previously in *Mecp2^−/y^* mice at up to 60 days post-natal, but the time course is not well established [22,23,29,30]. In the current study, we followed female subjects for longer than five months and found that motor function declines steadily through about four months of age, which may be consistent with the progressive decline with age observed in the late motor deterioration stage in patients with RTT. Interestingly, voluntary ambulatory behavior remained largely preserved throughout the study period; even when *Mecp2^−/x^* rats could barely remain on the rotarod, there was no difference of night time voluntary activity and only a moderate decline in diurnal activity, which may suggest that central structures initiating movement may not be affected. On the other hand, poor rotarod performance did correlate significantly with increased swing phase of the step cycle, suggesting that the increased time required to move the foot into the braking position may have led to instability and discoordination.

A curious feature of the motor dysfunction is the rostro-caudal asymmetry of the effect, where the forelimbs are disproportionately affected. This would seem to be consistent with prior reports in heterozygous *Mecp2*-deficient mice [30]. The *Mecp2*-308 female mice show defects in purposeful forelimb use as evidenced by impaired handling of nesting material, food and novel environment exploration [31]. Deficient forelimb movement and repetitive patterns is also a key feature of human RTT [32,33,34]. Hence, a greater deficit in forelimb motor function appears to be consistent across male mouse and female rat RTT models as well as in human subjects.

Somatosensory deficits are widespread in ASD [35,36] and abnormal sensory perception as well as indications of pain in RTT patients have been reported by parents and care-givers [16,17]. Nonetheless, it has been challenging to document the exact nature and progression of sensory deficits in patients, which is especially problematic in RTT owing to deficits in verbal communication abilities. Using *Mecp2^−/y^* rodent models, we and others found somatosensory deficits associated with *Mecp2* mutation which appears to be cell-autonomous to primary sensory neurons of the dorsal root ganglion [22,23]. Hence, deletion of *Mecp2* appears to result in altered transcription within the ganglion that directly affects sensory neurons through effects at the levels of the peripheral and central terminals [22]. Consistent with our findings in male *Mecp2^−/y^* rats, *Mecp2^−/x^* female rats showed marked hypersensitivity to mechanical and noxious cold stimuli [22] although the onset was marginally delayed (eight weeks vs. four weeks in *Mecp2^−/y^*) but sustained thereafter. In contrast to *Mecp2^−/y^* rats, thermal hypoalgesia was not observed in *Mecp2^−/x^* rats.

One question that remains unanswered is whether other behavioral deficits occur secondary to somatosensory disturbances. In light of the increased mechanical sensitivity associated with *Mecp2* deletion, it is possible that this could contribute to reduced motor performance. Hence, it is possible that the step cycle may be impacted by ‘guarding’, where animals preferentially spend less time in the propel phase where the increased force associated with propulsion could be perceived as painful. Similarly, while we know that sensory nociceptor [22] and low threshold tactile receptor [23] neuronal functions are altered, it is likely that other types of sensory function may also be disturbed. Accordingly, it is possible that proprioception may also be affected and, if so, this could contribute to alterations in step cycle and posture that occur in these animals. Further investigation will be necessary to elucidate the extent to which motor deficits occur secondary to sensory dysfunction.

This study also provides evidence that visceral perception is disrupted in this female RTT rat model. In human RTT patients, gastrointestinal and bowel related problems are prominent [20,37]. There is strong evidence that patients with RTT experience significant gastrointestinal dysmotility leading to gastroesophageal reflux, constipation, bloating, with resultant distress and frequent abdominal pain [37]. Our findings point to colonic hyposensitivity and/or hyporeflexia as a prominent component of the RTT rat model phenotype. Balloon distension of the colon resulted in a much-diminished abdominal muscular activation, which would be consistent with dysmotility. Visceral function and sensitivity are regulated by complex circuits involving intrinsic enteric neuronal plexuses as well as extrinsic contributions from the autonomic and nervous system and dorsal root ganglia [38,39,40,41,42]. Dysregulation of autonomic functions has been reported in RTT [43,44,45], although *Mecp2* is widely expressed in all neuronal and target cells such that the etiology of the visceral dysfunction remains uncertain. Nonetheless, the *Mecp2^−/x^* rat should prove to be a useful model in helping to define mechanisms of RTT visceral dysfunction.

Many pervasive developmental disorders including autism are associated with alterations in metabolism [25,26]. However, prior studies using mouse models failed to reveal a systematic trend in metabolic disruption [15]. This may be attributable to the short duration of studies, use of null male mice which are underweight and survive only to two months of age, and/or interactions between the mutation and the genetic background of the different strains. Some studies of *Mecp2* mouse models have reported altered body weight, with both increases and decreases observed and occurring in a potentially strain-specific manner [46,47,48]. *Mecp2* deletion restricted to the hypothalamus results in increased weight gain in association with increased food intake [46]. Our findings of increased weight gain in association with reduced food intake suggest that possibly other brain regions associated with satiety may be differentially affected in the different models, or that perhaps there is a more profound metabolic disturbance in the heterozygous female rat. Indeed, the *Mecp2^−/x^* rat also showed abnormal fat accumulation despite significantly lower food intake and largely comparable activity levels, consistent with severe metabolic dysfunction. This is also consistent with some missense and late truncating *Mecp2* mutations that give rise to a RTT variant known as the Zappella, where subjects are overweight [4,49]. Humans with RTT show elevated total cholesterol and LDL levels in plasma [50], and *Mecp2^−/y^* mice exhibit defective cholesterol metabolism in brain and liver [51]. It is unclear whether the increased body fat promotes a systemic pro-inflammatory state that may alter sensory neuron thresholds [52,53], thus contributing to the hypersensitivity we see in this model. It is noteworthy that both maternal fostering and genetic modifiers associated with different background strains have been shown to be strong contributors to behavioral and metabolic phenotype [54,55], and interactions with different mutations might also play a prominent role in determining the metabolic outcome. The molecular mechanisms underlying metabolic dysfunctions in RTT remain to be determined.

## 4. Materials and Methods

### 4.1. Animals

All animal experiments were conducted on a total of 24 female rats and were approved by the KUMC IACUC. Associated protocols used in this study will be provided upon request. The *MECP2* knockout rat was generated by a zinc finger nuclease mediated 71 bp deletion in exon 4 of *MECP2* gene (Product# TGRA6090, Sage Labs, Boyertown, PA, USA).

### 4.2. Timelines for Measurement

Table 4 outlines the frequency and time line (by animal age) for tests and measurements administered in this study.

### 4.3. Rotarod

Balance and coordination were tested using an accelerating rotarod (Med Associates Inc., St. Albans, Vermont, USA) with an initial speed of 4 rpm ramping to 40 rpm over 300 s. Rats were placed on the 70-mm diameter rod elevated 27 cm above the table. The accelerating program was initiated and the latency to fall off the rod was recorded and averaged from three independent trials at least 5 min apart.

### 4.4. Gait Analysis

Fully automated gait analysis was conducted with the DigiGait (Mouse Specifics, Framingham, MA, USA) treadmill at a walking speed of 8.5 cm/s. The DigiGait system records foot falls of the subject from the ventral aspect and then automates the analysis of over 40 parameters including brake, propel and stride time, coordination, and ataxia coefficient [21].

### 4.5. Somatosensory Behavior: Mechanical, Thermal and Cold Sensitivity

Mechanical sensitivity was analyzed using Siemens Weinstein monofilaments of known, graded force. Animals were placed in individual Plexiglas boxes on an elevated mesh table and allowed to acclimate for 20 min. Following acclimation, six monofilaments ranging from 2 to 15 g were applied five times to each hind paw at intervals of not less than 2 min. The number of positive responses, as observed by lifting or biting of the hind paw, was recorded. The calibrated force of the monofilament generating a 50% withdrawal response rate was recorded as the withdrawal threshold.

Thermal sensitivity was measured as withdrawal latency to a fixed 4.85-amp source directed to the paw in a Paw Thermal Stimulator (University of California, San Diego, CA, USA). The glass floor was maintained at 30.0 °C and 20 min of acclimation was allowed prior to testing. Light beam intensity was set at 4.85 amp and withdrawal latency defined as the time from stimulus onset until the automatic sensor turned the light off in response to paw lifting. Thermal withdrawal latencies of each hind paw were measured in triplicate, with a minimum of 3 min between trials, and were averaged.

Cold sensitivity was measured using a Cold Plate Analgesia Meter (IITC Life Science Inc., Los Angeles, CA, USA). Rats were acclimated for five min at 25 °C, and thermal plate temperature was decreased at a ramp rate of 10 °C per min. Upon observing overt nociceptive behavior such as lifting, biting or licking of the hind paws, the thermal plate was immediately returned to 25 °C. Testing was conducted twice with a 5–10 min interval between trials. The average temperature at which nociceptive behaviors were seen is reported as the withdrawal threshold.

### 4.6. Visceral Sensitivity

Rats were initially anesthetized with 4% inhaled isoflurane followed by continually-administered 1.5–3% inhaled isoflurane to maintain anesthesia. A small, catheterized (0.5 cm × 4 cm) latex or nitrile balloon was inserted into the distal colon. The rat was then put on a 37 °C heating pad under continuous 1.5% isoflurane anesthesia through nose cone and the balloon was distended for 20 s to 20, 40, 60 and 80 mm Hg (each pressure in triplicate with 4 min inter-trial intervals) and the electromyographic activity of the abdominal musculature were measured by thin silver electrode wires implanted acutely into the abdominal muscle on either side of the abdominal midline; the ground was attached to the tail. The VMR was quantified by measuring the area under the curve of the entire distension period divided by the duration of distension and expressed as a percentage of baseline EMG activity [24].

### 4.7. Food, Water and Activity Monitoring (Metabolic Cage)

Rats were placed in indirect calorimetry home cages (Sable Systems, Las Vegas, NV, USA) and were preconditioned prior to collection of data to eliminate effects of the novel environment. The gas from the cages is sampled each second for O_2_, CO_2_, and H_2_O content. Food, water and animal habitats are placed on weight sensors and movement of the subject is measured with external infrared beams. Fully automated software calculates activity (free ambulation) respiratory quotient, distance traveled, and others.

### 4.8. Body Mass Composition

Lean and fat mass was analyzed with the EchoMRI Body Composition Analyzer (EchoMRI LLC, Houston, TX, USA). Briefly, unanesthetized rats are placed in the analyzer for approximately 60 s and proprietary NMR-MRI technology is used to calculate fat, lean, free water and total water of the subject.

### 4.9. PBMNC Isolation, Staining and FACS

About 400–500 µL blood was collected from the tail vein of each *Mecp2^−/x^* rat into EDTA coated vials. The blood was diluted 1:1 in sterile PBS layered over Percoll and centrifuged. The buffy coat layer containing the peripheral blood mononuclear cells (PBMNCs) was collected and fixed by adding equal volume of 8% PFA (to make a final 4% concentration). The cells were washed thrice in PBS and then stained for 20–30 min on ice (wrapped in aluminum foil) with FITC conjugated Anti-CD3 antibody at 1:200 (BD Bioscience, San Jose, CA, USA) to label all T-cells. After a wash, primary anti-*Mecp2* (rabbit-anti, Cell Signaling Technology, Danvers, MA, USA) was added and incubated for 1 h. After washes, donkey anti-rabbit Cy3 (Jackson Immuno-research, West Grove, PA, USA) at 1:200 and incubated for 30 min. Following three washes, cells were FACS analyzed to determine the percentage of *Mecp2* expressing cells within the CD3 positive population.

### 4.10. Stastical Analysis

The statistical software SigmaPlot (Systat, San Jose, CA, USA) was used to perform analyses reported here. Comparisons of longitudinal measures of behavioral parameters were done by two way repeated measure ANOVA on ranks and using Holms-Sidak for post hoc comparisons. For comparison of two groups and single time point data, unpaired *t*-tests using Student Newman-Keuls were performed. In all cases *p <* 0.05 is reported to be significant. All data were reported as Mean ± SEM, unless stated otherwise.

## Figures and Tables

**Table 1:** Summary of statistical analyses for two-way repeated measure ANOVAs.

Test	Source of Variation	DF	F	P
Body Weight	Genotype	1	12.487	0.002
	Weeks	9	310.219	<0.001
	Genotype × Weeks	9	6.613	<0.001
Rotarod	Genotype	1	83.617	<0.001
	Weeks	9	16.738	<0.001
	Genotype × Weeks	9	8.143	<0.001
Von Frey	Genotype	1	21.737	<0.001
	Weeks	9	3.649	<0.001
	Genotype × Weeks	9	0.281	0.979
Cold Plate	Genotype	1	36.002	<0.001
	Weeks	9	2.266	0.021
	Genotype × Weeks	9	0.780	0.635
VMR	Genotype	1	6.514	0.021
	Pressure	3	33.405	<0.001
	Genotype x Pressure	3	4.600	0.007

**Table 2:** Metabolic indices measured by calorimetry during light and dark phases over a 48-h test period in wild type (WT) and heterozygous (Het) rats. VO2 = oxygen consumed as O2 mL/min. VCO2 = carbon dioxide produced as mL/min. RQ = Respiratory Quotient obtained as VCO2/VO2. All measurements are from a single rat per cage.

**Light**					
**Parameters**	**WT Mean**	**WT SEM**	**Het Mean**	**Het SEM**	***t*-Test *p*-Value**
Average Energy Expenditure	1.85	0.04	1.67	0.09	0.199
Total Energy Expenditure	25.96	0.56	23.43	1.27	0.199
Average VO2	6.41	0.14	5.88	0.31	0.26
Average VCO2	5.09	0.16	4.28	0.28	0.073
Average RQ	0.79	0.02	0.72	0.02	0.03
**Dark**					
**Parameters**	**WT Mean**	**WT SEM**	**Het Mean**	**Het SEM**	***t*-Test *p*-Value**
Average Energy Expenditure	2.29	0.03	2.01	0.12	0.138
Total Energy Expenditure	22.85	0.31	20.09	1.2	0.138
Average VO2	7.86	0.13	7.01	0.41	0.172
Average VCO2	6.42	0.11	5.3	0.38	0.064
Average RQ	0.82	0.02	0.75	0.02	0.054

**Table 3:** Summary of regression analyses performed among behavioral measurements in *Mecp2^−/x^* rats.

Parameters Compared	R^2^	Adjusted R^2^	F-Statistic vs. Constant Model	*p*-Value
Weight gain rate and day time food intake	0.0152	−0.108	0.124	0.734
Weight gain rate and night time food intake	0.0522	−0.0663	0.441	0.525
Weight gain rate and day time water intake	0.0405	−0.0794	0.338	0.577
Weight gain rate and night time water intake	5.73 × 10^−6^	−0.125	4.58 × 10^−5^	0.995
von Frey and Brake	0.195	−0.00572	0.972	0.38
von Frey and Propel	0.307	0.134	1.77	0.254
von Frey and Swing	0.0428	−0.196	0.179	0.694
Sensitivity (% withdrawal) to a 4 mg filament and brake	0.564	0.456	5.18	0.0851
Sensitivity (% withdrawal) to a 4 mg filament and Propel	0.514	0.393	4.23	0.109
Sensitivity (% withdrawal) to a 4 mg filament and Swing	0.0482	−0.19	0.202	0.676
Rotarod and Brake	0.204	0.00471	1.02	0.369
Rotarod and Propel	0.0255	−0.218	0.105	0.762
Rotarod and Swing	0.706	0.633	9.62	0.0362

**Table 4:** Behavioral testing schedule.

Test	Frequency of Measure	Age
Rotarod	Biweekly	Weeks 4–22
DigiGait	Once	Week 18
Somatosensory (mechanical, thermal, cold)	Biweekly	Weeks 4–22
Visceral sensitivity	Once	Week 18
Metabolic (food, water, activity)	Once	Week 18
Body mass composition	Once	Week 18

## Abbreviations

RTT	Rett Syndrome
ASD	Autism spectrum disorder
*MECP2*	Methyl CpG-binding Protein 2
WT	Wild type
PBMNC	Peripheral blood mononuclear cell
EMG	Electromyography
VMR	Visceromotor reflex
